# A comparative study of the characterization of miR-155 in knockout mice

**DOI:** 10.1371/journal.pone.0173487

**Published:** 2017-03-09

**Authors:** Dong Zhang, Yongchun Cui, Bin Li, Xiaokang Luo, Bo Li, Yue Tang

**Affiliations:** Chinese Academy of Medical Sciences, Peking Union Medical College, National Centre for Cardiovascular Disease, Fuwai Hospital, State Key Laboratory of Cardiovascular Disease, Beijing Key Laboratory of Pre-Clinical Research and Evaluation for Cardiovascular Implant Materials, Animal Experimental Centre, Beijing, China; Universidade Federal do Rio de Janeiro, BRAZIL

## Abstract

miR-155 is one of the most important miRNAs and plays a very important role in numerous biological processes. However, few studies have characterized this miRNA in mice under normal physiological conditions. We aimed to characterize miR-155 in vivo by using a comparative analysis. In our study, we compared miR-155 knockout (KO) mice with C57BL/6 wild type (WT) mice in order to characterize miR-155 in mice under normal physiological conditions using many evaluation methods, including a reproductive performance analysis, growth curve, ultrasonic estimation, haematological examination, and histopathological analysis. These analyses showed no significant differences between groups in the main evaluation indices. The growth and development were nearly normal for all mice and did not differ between the control and model groups. Using a comparative analysis and a summary of related studies published in recent years, we found that miR-155 was not essential for normal physiological processes in 8-week-old mice. miR-155 deficiency did not affect the development and growth of naturally ageing mice during the 42 days after birth. Thus, studying the complex biological functions of miR-155 requires the further use of KO mouse models.

## Introduction

microRNAs (miRNAs) are a type of small noncoding RNA that regulates gene expression at the post-transcriptional level [1]. microRNA-155 (miR-155) is one of the most important miRNAs and plays an important role in various biological processes, including immunity [2,3],inflammation [4,5], viral infections [6], cancer [7,8] and cardiovascular disease [9,10]. The homeostasis of inflammation and inflammatory cells is closely related to miR-155 expression and plays a critical role in T cell and B cell development [11,12]. Recently, increasing numbers of studies have demonstrated the broad potential of miR-155 for use in further research, although few studies have characterized this miRNA in mice under normal physiological conditions.

In our previous experiments, we established a miR-155 knock-out (KO) mouse model using the CRISPR/Cas9 system and established a homozygous KO mouse line (S1–S3 Figs). We aimed to utilize this mouse model to identify the biological functions of miR-155. Based on preliminary research, we compared miR-155 knockout (KO) mice with C57BL/6 wild type (WT) mice to characterize miR-155 under normal physiological conditions.

## Materials and methods

### Animals

All animal procedures that included the use of an anaesthetic agent were approved by the Animal Ethics Committee of the Fuwai Hospital of Peking Union Medical College, and all experiments were conducted in strict accordance with the National Institutes of Health Guide for the Use of Laboratory Animals. All efforts were made to minimize suffering. Our experiments were conducted using KO and WT mice. The genotypes of all mice were distinguished by agarose gel electrophoresis, which ensured that the miR-155 had indeed been knocked out in the KO mice (S4 Fig). These mice were obtained from the animal centre in Fuwai Hospital of Peking Union Medical College (Beijing, China). The animals were housed under standard conditions with a 12 h light/dark cycle and adequate water and food. The mice were anaesthetized using chloral hydrate and isoflurane.

### Growth curve and groups

Twenty parents (10 male and 10 female mice, aged 8 weeks) were randomly selected from both the WT and KO mouse populations. All mice were monogamously mated. After natural delivery, reproductive performance parameters were tested, and growth curves were constructed.

Next, the offspring were randomly divided into four groups as follows: control groups (WT male and female groups) and model groups (KO male and female groups). Each group contained 10 mice (each weighing from 20 to 25 g and aged 8 weeks).

### Abdominal ultrasound and echocardiography

To standardize the ultrasonic inspections and measurements, only mice with heart rates within the range of 400–600 beats per min (bpm) during anaesthesia were included in the analysis [13].

Ultrasonic inspections and measurements of the kidney, liver, and heart were completed using the VisualSonics Vevo 2100 ultrasound system (VisualSonics, Toronto, ON, Canada). First, the mice were anaesthetized with an intraperitoneal injection of 20 mg/kg of chloral hydrate (2.5% in normal saline) and maintained under anaesthesia with 2.6% isoflurane through a nose cone during the operation. The mice were placed in the supine position on a heated platform with ECG leads. Depilatory paste was used to remove fur in the region of detection, and medical ultrasound gel was used to ensure optimal imaging. Using ultrasound guidance, the organ parameters were identified and recorded. All examinations were performed by one experienced operator, and all measurements were repeated three times at the same site [14].

### Biochemical detection and blood routine examination (blood RT)

The mice were introduced into the anaesthetic chamber and sedated with 5% isoflurane for 1 min. Retro-orbital blood samples acquired to determine the haematological indices were stored in tubes containing the anticoagulant ethylenediaminetetraacetic acid (EDTA) [15]. The serum levels of alanine aminotransferase (ALT), aspartate transaminase (AST), total protein (TP), albumin (ALB), globulin (GLB), albumin/globulin (A/G), creatinine (CRE), blood urea nitrogen (BUN), and blood glucose (GLU) were determined for the mice. An automated haematology analyser was used to analyse haematological parameters including the following: white blood cells (WBC), red blood cells (RBC), haemoglobin (HGB), haematocrit (HCT), mean corpuscular volume (MCV), mean corpuscular haemoglobin (MCH), mean corpuscular haemoglobin concentration (MCHC), red cell distribution width (RDW), platelet count (PLT), thrombocytocrit (PCT), mean platelet volume (MPV), platelet distribution width (PDW), lymphocytes (LYM), monocytes (MON), neutrophils (NEUT), eosinophils (EOS), and basophils (BAS).

### Organ weight and histopathological analysis

At the end of the study, all mice were euthanized for necropsy (cervical dislocation method). The weights of the vital organs and the organ-to-body weight ratios were determined. Selected organs were weighed and recorded, and histopathological examinations of tissue slices from the liver, kidney, spleen, heart, intestine, lung and brain were completed.

The excised organs were cut into transverse slices of approximately 2 mm and were fixed in 4% paraformaldehyde. After embedding in paraffin, many transverse sections were obtained and then stained using haematoxylin and eosin (HE).

Histological changes in the main organs (the liver, kidney, spleen, heart, intestine, lung and brain) were graded on a scale of 0–9 based on the degree of architecture degeneration, inflammation and necrosis [16].

The scoring system used to assess the histological changes was as follows. A score of 0 indicated normal architecture and histology. For A (architecture), a score of 1 indicated that architecture degeneration was observed in ≤20% of the cells, 2 indicated that architecture degeneration was observed in 21–50% of the cells, and 3 indicated that the architecture degeneration was observed in more than 50% of the cells. For I (inflammation), 1 indicated minimal inflammation, 2 indicated small localized inflammation, and 3 indicated diffuse inflammation; for N (necrosis), 1 indicated necrotic foci in a few cells at one location, 2 indicated necrotic foci at multiple locations, and 3 indicated diffuse necrosis.

### Statistical analysis

The data are shown as the mean ± standard deviation. All parameters were compared between the control groups and the model groups using one-way analysis of variance (ANOVA). P-values less than 0.05 were considered statistically significant. GraphPad Prism 5 software (San Diego, CA, USA) was used for the statistical analyses.

## Results

### Reproductive performance and growth curves

The KO mice were developed in an inbred line. As required, mice with stable inheritance, strong reproductive abilities and optimal maternity were chosen and monogamously mated.

As summarized in Table 1, a total of 56 mice (23 females and 33 males) were born in the model groups, and61 mice (27 females and 34 males) were born in the control groups. The two groups showed no significant differences in litter sizes, weights, lengths, or weaning rates (P > 0.05).

**Table 1:** Summary of reproductive performance.

Strain	No. of offspring	Pups/ Litter	Weight of NM^#^ (g)	Length of NM^#^ (mm)	Weight of WM^#^ (g)	Length of WM^#^ (mm)	Male:Female	Weaningrate(%)
KO	n = 56	5.60±0.31	1.246±0.010	39.79±0.73	9.36±0.25	119.02±0.89	1.43:1	91.07
WT	n = 61	6.10±0.28	1.254±0.014	38.71±0.74	9.84±0.27	121.00±0.90	1.26:1	93.44

Note

# NM = neonatal mice; WM = weaned mice; the values represent the mean ±standard deviation. One-way analysis of variance and pairwise comparison were performed; and P > 0.05 indicates no significant change compared with the control.

**Fig 1:**
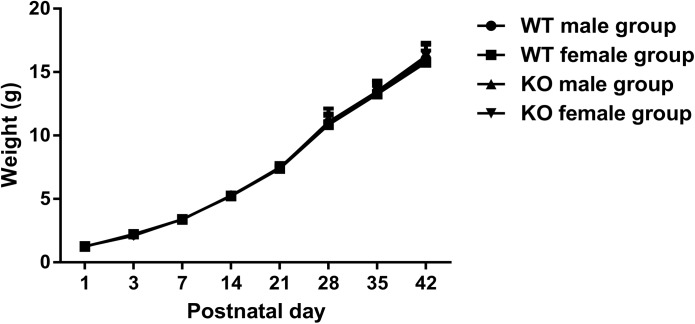
Comparative growth curves. Line chart showing the weight gain of the control and model groups from postnatal day 1 to postnatal day 42.

During the postnatal period, the weight of each neonatal mouse was recorded to generate growth curves. As summarized in Table 2, no significant difference in weight gain was found between the control and model groups (P > 0.05). No significant differences were observed in the growth curves (Fig 1).

**Table 2:** Changes in body weight.

Age	Body weight (g)			
	WT		KO	
	Male (n = 34)	Female (n = 27)	Male (n = 33)	Female (n = 23)
1d	1.27±0.01	1.25±0.01	1.25±0.01	1.28±0.01
3d	2.19±0.03	2.22±0.04	2.24±0.03	2.11±0.03
7d	3.40±0.04	3.40±0.04	3.38±0.04	3.41±0.05
14d	5.27±0.05	5.23±0.05	5.29±0.06	5.26±0.06
21d	7.48±0.06	7.38±0.05	7.44±0.07	7.51±0.06
28d	11.04±0.19	10.85±0.17	11.09±0.18	10.88±0.14
35d	13.47±0.13	13.26±0.13	13.49±0.15	13.50±0.14
42d	16.08±0.21	15.76±0.18	15.98±0.22	16.28±0.18

Note: The values represent the mean ±standard deviate on. One-way analysis of variance and pairwise comparisons were performed, and no significant differences between KO and WT mice were observed (P > 0.05).

### Ultrasonographic appearance of the mouse kidney and liver

Ultrasound examinations were used to evaluate the development and condition of the abdominal organs in the mice, and the liver and kidney parameters were measured and recorded. The indices used for evaluating the kidney included the longitudinal kidney, transverse kidney, renal artery diameter (RA Diam), renal artery end diastolic velocity (RAEDV), renal artery peak systolic velocity (RAPSV), and renal artery resistance index (RARI). The indices used for evaluating the liver included the longitudinal liver, transverse liver, portal vein diameter (PV Diam), velocity time integral (VTI), mean velocity (Mean Vel), mean grad, peak velocity (Peak Vel), and peak grad.

**Fig 2:**
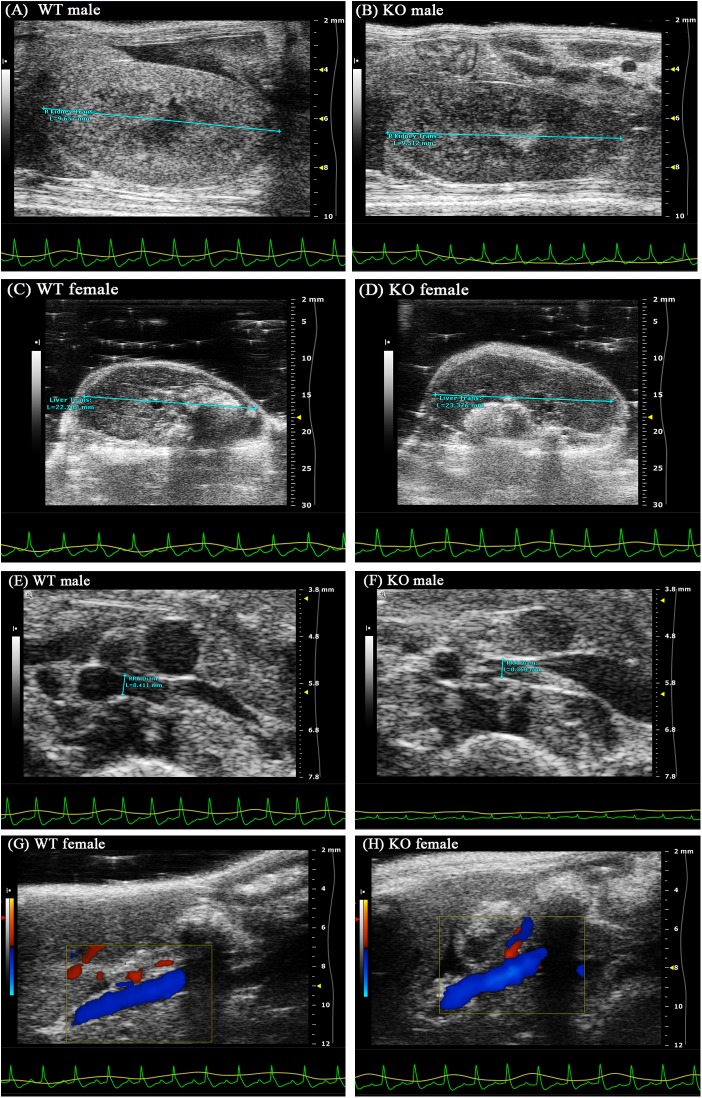
Representative parameters of the kidney and liver. The liver and kidney parameters were measured and recorded based on ultrasound examinations. (A and B) Transverse kidney; (C and D) Transverse liver; (E and F) RA diameter; and (G and H) PV visualization. The parameters showed no significant differences in any of the evaluated indices. (Mice were 8 weeks old.)

These evaluations showed that all animals were physiologically normal. Ultrasound examination did not show tumours, hydronephrosis, cysts or other lesions in any of the groups. The inspection and measurement parameters showed no significant differences in any of the evaluation indices in the mice in the control and model groups (Fig 2). Specific parameters are presented in Table 3.

**Table 3:** Abdominal ultrasound parameter data.

Parameter (unit)	WT		KO	
	Male	Female	Male	Female
Longitudinal Kidney (mm)	5.85±0.11	5.80±0.13	5.81±0.19	5.77±0.15
Transverse Kidney (mm)	9.42±0.28	9.14±0.37	9.41±0.54	9.08±0.51
RA Diam (mm)	0.41±0.03	0.38±0.01	0.40±0.01	0.38±0.02
RAEDV (mm/s)	161.39±8.53	154.92±9.12	156.47±9.82	141.66±8.34
RAPSV (mm/s)	605.52±40.29	599.64±34.72	590.31±49.18	594.17±51.19
RARI	0.74±0.03	0.71±0.04	0.77±0.04	0.72±0.04
Longitudinal Liver (mm)	16.27±0.32	16.36±0.33	16.67±0.35	16.60±0.35
Transverse Liver (mm)	21.53±0.66	21.92±0.94	21.26±1.08	22.18±0.75
PV Diam (mm)	1.43±0.05	1.48±0.09	1.50±0.07	1.51±0.05
VTI (mm)	56.96±4.49	55.30±4.20	53.79±4.31	60.93±5.29
Mean Vel (mm/s)	149.03±7.10	146.30±7.21	147.58±6.45	152.60±8.95
Mean Grad (mmHg)	0.12±0.01	0.10±0.01	0.11±0.01	0.12±0.02
Peak Vel (mm/s)	193.47±7.07	183.17±6.99	186.17±6.54	184.27±8.21
Peak Grad (mmHg)	0.16±0.01	0.13±0.01	0.14±0.02	0.16±0.01

Note: The values represent the mean ± standard deviation from n = 10.One-way analysis of variance and pairwise comparison were performed, and P > 0.05 indicates no significant change compared with the respective control.

### Echocardiography data analysis

Cardiac functions were evaluated by echocardiography. For more effective inspection and measurement, the sweep speed, depth, and focus were adjusted to acquire the best images. The left ventricular end-diastolic diameter (LVEDD), left ventricular end-systolic diameter (LVESD), left ventricular end-diastolic volume (LVEDV), left ventricular end-systolic volume (LVESV), left ventricular posterior wall (LVPW) diastole and shrink (D and S), left ventricular anterior wall (LVAW) D and S, interventricular septum (IVS) depth and left atrium (LA) depth were measured and recorded using echocardiography. Left ventricular stroke volume (LVSV), left ventricular ejection fraction (LVEF), left ventricular fractional shortening (LVFS), cardiac output (CO), and LV mass (LVM) were calculated from the above parameters.

**Fig 3:**
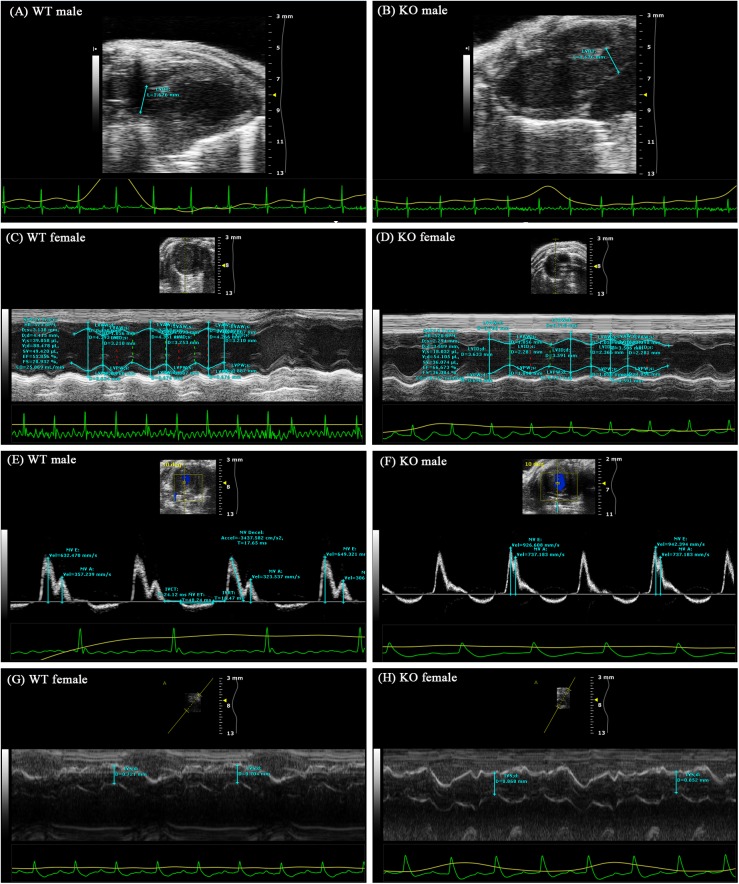
Representative echocardiography parameters. Cardiac functions were evaluated by echocardiography. (A and B) Length of the left ventricular outflow tract (LVOT); (C and D) Measurement and recording of the left ventricular short axis view; (E and F) Ventricular diastolic function detection: the ratio of the peak E and A wave velocities of the mitral valve rheography (E/A); and (G and H) IVS depth. The parameters showed no significant differences in the main evaluation indices. (Mice were 8 weeks old.).

The results showed that all animals were in optimal condition and that none of the animals had lesions or other cardiac diseases. The measured and calculated parameters showed that only the WT male group had an obviously lower LVESD than the KO male group (P < 0.05), and no significant differences were observed in the other parameters in any of the groups (P > 0.05) (Fig 3). In particular, no significant differences between groups were found in the LVEF, IVS depth, LVDD and CO. Specific parameters are illustrated in Table 4.

**Table 4:** Cardiac ultrasound parameter data.

Parameter (unit)	WT		KO	
	Male	Female	Male	Female
Heart Rate (beats per min)	521.3±13.42	537.9±14.13	528.8±12.39	515.8±14.23
IVS depth (mm)	0.73±0.03	0.73±0.02	0.74±0.02	0.72±0.02
LA depth (mm)	1.63±0.10	1.64±0.10	1.59±0.08	1.67±0.10
LVEDD (mm)	3.53±0.22	3.44±0.19	3.48±0.15	3.42±0.17
LVESD (mm)	1.58±0.05*	1.96±0.10	1.91±0.07*	1.96±0.08
LVEDV (μl)	53.82±1.94	55.15±2.08	54.72±1.92	55.55±2.20
LVESV (μl)	17.81±0.91	19.1±0.68	18.05±1.08	18.01±0.71
LVSV (μl)	35.79±1.55	36.96±1.60	35.45±1.30	35.79±1.54
LVEF (%)	69.57±1.34	68.32±2.30	65.36±2.15	63.73±1.50
LVFS (%)	40.58±1.66	39.35±1.55	39.10±1.64	40.13±2.10
CO (mL/min)	21.67±0.78	21.66±0.62	20.94±0.80	21.95±1.07
LVAW(D) (mm)	0.73±0.02	0.76±0.02	0.71±0.03	0.74±0.01
LVAW(S) (mm)	1.28±0.03	1.23±0.02	1.21±0.01	1.22±0.02
LVPW(D) (mm)	0.71±0.03	0.74±0.02	0.69±0.03	0.65±0.03
LVPW(S) (mm)	1.26±0.05	1.22±0.02	1.15±0.02	1.20±0.02
LV Mass (mg)	68.32±2.26	67.15±2.41	67.42±2.48	66.91±2.61

Note: The values represent the mean ± standard deviation from n = 10. One-way analysis of variance and pairwise comparison were performed, and

* P < 0.05 indicates a significant change compared with the control.

### Blood physiological values

The haematological indices of the blood physiological values were used to evaluate mouse homeostasis. As summarized in Table 5, the blood physiological values of the KO mice were all in the normal range, as were those of the control groups, and no significant differences were detected between the control and model groups (P > 0.05). In particular, no significant differences were observed in the WBC, RBC, HGB and PLT counts between the groups.

**Table 5:** Blood physiological values of the control and model groups.

Parameter (unit)	WT		KO	
	Male	Female	Male	Female
WBC(×10^9^/L)	7.11±0.76	7.37±0.53	6.830±0.55	7.52±0.40
RBC (×10^12^/L)	8.79±0.54	8.61±0.42	8.16±0.47	8.40±0.50
HGB (g/L)	134.90±3.99	126.90±3.66	127.20±4.66	129.80±3.96
HCT (%)	44.67±1.20	42.49±1.12	42.50±0.99	42.81±0.59
MCV (fL)	47.26±0.56	47.91±0.73	47.65±0.40	47.57±0.28
MCH (pg)	14.42±0.19	14.59±0.15	14.57±0.16	14.75±0.18
MCHC (g/L)	300.40±2.21	300.10±2.29	299.20±1.43	300.50±1.71
RDW (%)	13.92±0.42	13.78±0.28	13.92±0.42	13.78±0.28
PLT (×10^9^/L)	504.60±77.45	520.90±61.47	471.90±60.37	454.00±53.73
PCT (%)	0.27±0.04	0.22±0.04	0.25±0.03	0.24±0.02
MPV (fL)	4.47±0.28	4.37±0.27	4.29±0.20	4.61±0.22
PDW (%)	16.50±0.14	16.51±0.21	16.06±0.20	16.14±0.21
LYM (×10^9^/L)	3.68±0.49	4.24±0.36	4.13±0.32	4.03±0.36
MON (×10^9^/L)	0.21±0.03	0.19±0.04	0.20±0.03	0.20±0.03
NEUT(×10^9^/L)	2.67±0.31	2.91±0.33	2.88±0.30	2.91±0.31
EOS (×10^9^/L)	0.12±0.04	0.11±0.04	0.12±0.03	0.12±0.03
BAS (×10^9^/L)	0.00±0.00	0.00±0.00	0.00±0.00	0.00±0.00
LYM%	53.71±1.11	54.26±1.93	51.15±1.31	50.64±1.02
MON%	2.59±0.48	2.30±0.47	2.47±0.45	2.39±0.41
NEUT%	42.42±1.26	36.28±4.28*	44.95±1.22	45.65±0.97*
EOS%	1.28±0.35	1.17±0.38	1.43±0.38	1.41±0.36
BAS%	0.00±0.00	0.00±0.00	0.00±0.00	0.00±0.00

Note: The values represent the mean ± standard deviation from n = 10. One-way analysis of variance and pairwise comparison were performed, and

* P < 0.05 indicates a significant change compared with the control.

The cytological classification counts were also evaluated. Only the KO female group had an obviously higher NEUT % than the WT female group (P < 0.05). However, increased NEUT counts were not observed among the other groups. No significant differences were found in LYM, MON or EOS (P > 0.05) among the groups, and all groups were within the normal range.

### Serum biochemical values

Biochemical detection was used to evaluate the functional status of the relevant organs. As summarized in Table 6, the serum biochemical values of the model groups were all within the normal range, as were those of the control groups, and no significant differences were detected between the control and model groups (P > 0.05). In particular, no significant differences existed among the groups in the ALT, AST, CRE, BUN and GLU levels.

**Table 6:** Serum biochemical values of the control and model groups.

Parameter (unit)	WT		KO	
	Male	Female	Male	Female
ALT (IU/L)	46.40±2.86	50.70±1.75	46.40±1.56	51.20±2.00
AST(IU/L)	196.30±17.41	220.60±18.76	193.20±15.70	210.00±8.85
TP (g/L)	46.77±1.33	50.41±1.03	49.46±0.89	49.01±0.89
ALB (g/L)	28.17±0.77	31.40±0.97	29.69±0.52	29.82±0.44
GLB (g/L)	18.60±0.75	20.01±0.84	19.77±0.49	19.19±0.61
A/G	1.53±0.05	1.54±0.06	1.51±0.03	1.57±0.04
CRE (μmol/L)	8.40±0.72	8.40±1.06	9.60±0.60	9.20±0.84
BUN (mmol/L)	9.13±0.27	9.16±0.38	9.70±0.41	9.89±0.41
GLU (mmol/L)	7.48±0.20	7.21±0.29	7.36±0.30	7.39±0.27

Note: The values represent the mean ± standard deviation from n = 10. One-way analysis of variance and pairwise comparison were performed, and P > 0.05 indicates no significant change compared with the respective control.

The results showed that almost all of the haematological and biochemical parameters were within the normal range. No significant differences were detected between the control and model groups. These data collectively indicated that all the animals were in good condition and that the miR-155 knockout did not cause any significant changes in the mice.

### Organ weight and weight ratio

At the end of the experiment, the weights of the organs and the organ-to-body weight ratios were determined to evaluate the development of the major organs. As summarized in Table 7, the WT male group had an obviously higher brain weight than the KO male group (P < 0.05). For the other parameters, no significant differences were observed among the groups (P > 0.05). The results showed normal growth and development of the major organs in both the control and model groups.

**Table 7:** Organ weights and ratios in the control and model groups.

Parameter (unit)	WT		KO	
	Male	Female	Male	Female
Weight (g)	21.59±0.39	21.47±0.34	21.67±0.34	21.52±0.35
Heart (g)	0.123±0.002	0.119±0.002	0.120±0.002	0.117±0.001
Lung (g)	0.160±0.001	0.152±0.002	0.154±0.002	0.154±0.001
Liver (g)	0.981±0.016	0.955±0.023	0.984±0.011	0.956±0.023
Spleen (g)	0.097±0.003	0.088±0.002	0.097±0.002	0.090±0.003
Brain (g)	0.415±0.003*	0.402±0.003	0.402±0.003*	0.397±0.003
Kidney (g)	0.281±0.003	0.271±0.005	0.278±0.005	0.275±0.004
Intestine (cm)	48.32±0.10	47.98±0.07	48.12±0.08	48.10±0.11
Heart/Body (%)	0.57±0.008	0.553±0.005	0.551±0.005	0.547±0.007
Lung/Body (%)	0.739±0.011	0.711±0.009	0.709±0.007	0.715±0.007
Liver/Body (%)	4.553±0.065	4.447±0.062	4.546±0.045	4.441±0.072
Spleen/Body (%)	0.447±0.012	0.408±0.007	0.450±0.006	0.420±0.011
Brain/Body (%)	1.924±0.028	1.875±0.022	1.866±0.015	1.848±0.019
Kidney/Body (%)	1.302±0.016	1.265±0.022	1.281±0.018	1.277±0.011

Note: The values represent the mean ± standard deviation from n = 10. One-way analysis of variance and pairwise comparison were performed, and

* P < 0.05 indicates a significant change compared with the control.

### Histopathological examination

**Fig 4:**
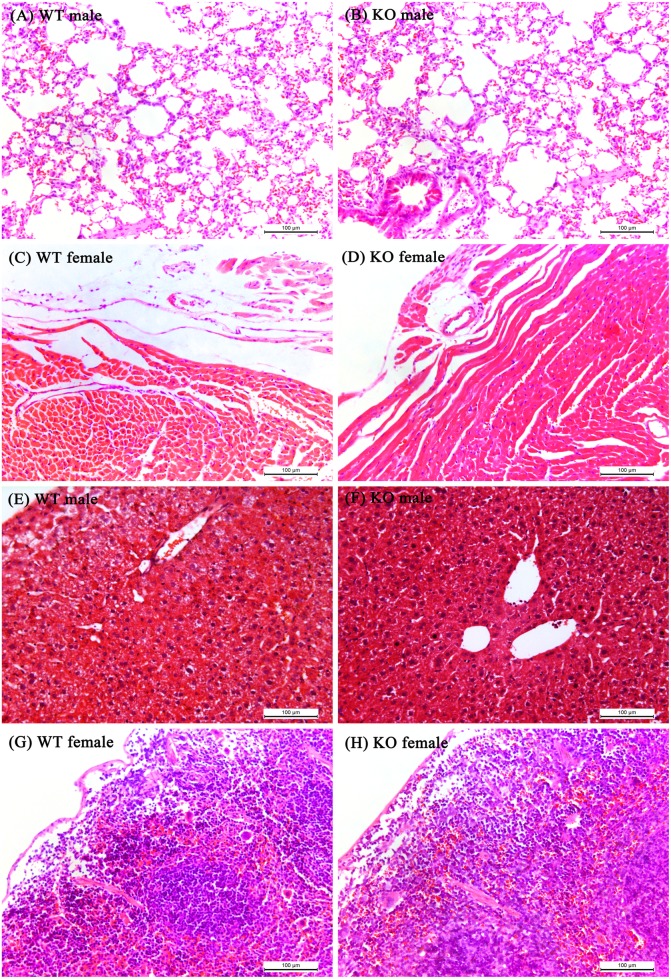
Representative photomicrographs of the vital organs. Sections fixed with paraformaldehyde were stained with haematoxylin and eosin (HE). Slides were observed with a light microscope (200×), and representative photomicrographs of sections of (A and B) lungs, (C and D) hearts, (E and F) livers, and (G and H) spleens from both the model and control groups are shown. No significant differences were observed for any of the evaluation indices. (Mice were 8 weeks old.).

Histopathological examinations of the major organs, including the liver, kidney, spleen, heart, intestine, lung and brain, were conducted to complete the final evaluation. The macroscopic examinations did not show any significant changes in the vital organs compared with the control groups, indicating that the miR-155 knockout had a minimal influence on mouse development. Next, microscopic examinations of selected vital organs were performed. The scoring of the pathological changes is presented in Table 8. The results revealed no histological changes between the control and model groups. Representative photomicrographs of the vital organs are shown in Fig 4.

**Table 8:** Summary of the histological scores.

Groups	Histological score^#^									
	0	Architecture			Inflammation			Necrosis		
		A1	A2	A3	I1	I2	I3	N1	N2	N3
**Liver**										
WT male group	9	0	0	0	1	0	0	0	0	0
WT female group	10	0	0	0	0	0	0	0	0	0
KO male group	9	1	0	0	0	0	0	0	0	0
KO female group	10	0	0	0	0	0	0	0	0	0
**Kidney**										
WT male group	10	0	0	0	0	0	0	0	0	0
WT female group	9	1	0	0	0	0	0	0	0	0
KO male group	9	1	0	0	0	0	0	0	0	0
KO female group	9	0	0	0	1	0	0	0	0	0
**Spleen**										
WT male group	9	0	0	0	1	0	0	0	0	0
WT female group	10	0	0	0	0	0	0	0	0	0
KO male group	10	0	0	0	0	0	0	0	0	0
KO female group	9	1	0	0	0	0	0	0	0	0
**Intestine**										
WT male group	9	1	0	0	0	0	0	0	0	0
WT female group	10	0	0	0	0	0	0	0	0	0
KO male group	9	1	0	0	0	0	0	0	0	0
KO female group	10	0	0	0	0	0	0	0	0	0
**Lung**										
WT male group	9	1	0	0	0	0	0	0	0	0
WT female group	10	0	0	0	0	0	0	0	0	0
KO male group	9	1	0	0	0	0	0	0	0	0
KO female group	10	0	0	0	0	0	0	0	0	0
**Brain**										
WT male group	10	0	0	0	0	0	0	0	0	0
WT female group	9	0	0	0	1	0	0	0	0	0
KO male group	9	1	0	0	0	0	0	0	0	0
KO female group	10	0	0	0	0	0	0	0	0	0
**Heart**										
WT male group	8	2	0	0	0	0	0	0	0	0
WT female group	9	0	0	1	0	0	0	0	0	0
KO male group	9	1	0	0	0	0	0	0	0	0
KO female group	9	1	0	0	0	0	0	0	0	0

Note

# The numeral in each row indicates the number of mice with a particular histological score.

Many organs showed a few scattered areas of degeneration and some inflammation. These phenomena were observed in all mice in both the control and model groups. Therefore, these areas of degeneration were not considered to indicate significant changes. However, almost all organs were found to have normal architecture, and no significant histological changes were observed in any of the mice.

## Discussion

miRNAs play important roles in biological processes by regulating mRNA expression or modifying mRNA structures [17,18]. For example, miR-155 serves very important biological functions. miR-155 affects the genesis and development of fibrosis, autophagy and inflammatory reactions [19]. Recently, miR-155 overexpression was demonstrated in several immunological diseases, including rheumatic disease [20–22], multiple sclerosis [23,24], and systemic lupus erythematosus [25,26].Numerous studies have shown that miR-155 is involved in a variety of biological processes, and we presume that this miRNA may play a vital role in the processes of organismal growth and development. Therefore, we used miR-155 gene knockout mice to characterize the function of miR-155 in mice.

In our study, we used many types of technology and methods, including a reproductive performance assessment, growth curve, ultrasonic estimation, haematological examination, and histopathological analysis, to evaluate growth and development in the control and model groups. The results showed no significant differences in the main evaluation indices in any of the groups. The KO mice exhibited almost no significant differences from the wild-type mice in any of the evaluation indices. Thus, miR-155 deficiency was shown to be minimally associated with growth and development in mice, and we found that miR-155 was a non-essential factor in mice under normal physiological conditions.

Therefore, the findings of our study are inconsistent with our preliminary assumption. At the beginning of the experiment, we presumed that miR-155 deficiency might produce at least a slight change in the function and structure of the mouse because a large number of studies have reported that miR-155 had a relatively wide range of biological characteristics; however, at the end of the study, we obtained the opposite result.

After the experiment, we referred to a large number of studies published in recent years and found that numerous miR-155-KO mice have been used in relevant experimental studies. A new study by Christmann RB [27] found that miR-155 was closely associated with lung fibrosis. A study by Seok HY [28] showed that a loss of miR-155 protected the heart from pathological cardiac hypertrophy. Gaudet AD and Fonken LK [29] found that miR-155 deletion in female mice prevented diet-induced obesity. Numerous experimental studies have shown that miR-155 plays a very important role in many biological processes.

However, we summarized the related studies and found that the miR-155-KO mice in these studies were modelled in various ways (i.e., drugs or operations). For example, the mice were exposed to bleomycin [30], underwent a transverse aortic constriction (TAC) operation [31], or were fed a controlled diet [32]. Detailed information is presented in Table 9.

**Table 9:** Summary of pertinent miR-155(-/-) mouse modelling studies.

Author	Pub. date	Journal	Model processing
Jab KA.et al.	2016 Jul	PLoS One.	Macrophages activated by M1
Wang W.et al.	2016 Jul	AJP Lung Cell Mol Physiol.	Bone marrow transplantation experiments
Bala S.et al.	2016 Jun	J Hepatol.	Lieber DeCarli diet
Zhang A.et al.	2016 May	J Heart Lung Transplant.	Heterotopic murine heart transplantation
Zhou S.et al.	2016 Apr	Arthritis Rheumatol.	Intraperitoneal injection of pristane
Gaudet AD.et al.	2016 Mar	Sci Rep.	Feed with controlled diet
Thome AD.et al.	2016 Feb	J Neurosci.	Injected with AAV2 virus (Stereotaxic surgery)
Yan Q.et al.	2016 Feb	Sci Rep.	Bleomycin
Yan L.et al.	2015 Sep	J Biol Chem.	Oxygen-induced retinopathy
LocRB.et al.	2015 Aug	PLoS One.	Borrelia burgdorferi-induced Lyme arthritis
LiFan L.et al.	2015 Jul	Immunity.	Retroviral transduction
Chen S.et al.	2015 Jul	Blood.	Haematopoietic cell transplantation (allo-HCT)
Curtis AM.et al.	2015 Jun	Proc Natl Acad Sci U S A.	Lipopolysaccharide (LPS)
Iwai H.et al.	2015 May	Tuberculosis (Edinb).	Infected with Mycobacterium tuberculosis Erdman
Lin X.et al.	2015 Apr	Inflammation.	Established hyperglycaemia-induced nephropathy
Singh UP.et al.	2014 Nov	Immunology.	Acute experimental colitis model
Pel KL.et al.	2014 Oct	Toxicol Sci.	Cisplatin
Lippai D.et al.	2014 Aug	Alcohol Clin Exp Res.	Ethanol containing Lieber-DeCarli
Solingen C.et al.	2014 Jun	J Cell Mol Med.	Wound healing
Seok HY.et al.	2014 May	Circ Res.	Transverse aortic constriction (TAC) operation
Sin SH.et al.	2013 Nov	J Virol.	Kaposi's sarcoma-associated herpesvirus (KSHV)
Yu F.et al.	2013 Aug	Mol Cancer Res.	Bone marrow transplantation
Clare S.et al.	2013 Mar	Infect Immun.	Mucosal Citrobacter rodentium infection
Huffaker TB.et al.	2012 Dec	Cell Rep.	Interferon γ (IFNγ)

Next, we found that few studies investigated miR-155 in mice under normal physiological conditions. To the best of our knowledge, our study is the first to report the characterization of miR-155 in naturally ageing mice using a comparative analysis. Our study presents a precise report of miR-155 in mice under normal physiological conditions. Based on the current study, we believe that a lack of miR-155 does not affect the normal physiological development and growth processes during the 42 days after birth, although miR-155 deficiency may strongly influence organismal physiology under pathological or malignant conditions.

The study of phenotypic features of the KO mouse is the basis for further studies characterizing miR-155. Although no obvious phenotype was observed in our research, the study of normal physiological conditions in the KO mouse is an important supplement to correlational studies about the biological characteristics and mechanism in various modelling processes. Our experimental results will provide a reference for further modelling and data analysis. Modelling of KO mice has been confirmed to be necessary to study the complex biological processes associated with miR-155.In subsequent experiments, we will further explore the characterization and functions of miR-155, especially the influence of miR-155 on cardiac functions.

## Conclusions

Using a comparative analysis, we found that miR-155 was a non-essential factor in normal physiological processes in 8-week-old mice. During the first 42 days after birth, miR-155 deficiency did not affect the development or growth of naturally ageing mice. Modelling with KO mice is necessary to study the complex biological processes of miR-155.

## Supporting information

S1 Fig Identification and targeting construction of miR-155 in mouse. miR-155 is encoded by the miR-155 host gene (HG). The miR-155 HG is processed into two pre-microRNAs (miR-155-5p and miR-155-3p). (A) This gene is composed of three exons, spans 13,024 bp, and encodes a 1500-bp non-coding primary-miRNA (pri-miRNA). Pre-miR-155 is matured from the pri-miRNA transcript. The location of pre-miR-155 is indicated by the orange box. (B) Pre-miR-155 includes a base-paired stem loop. The miR-155-5p sequence is shown in green, and the miR-155-3p sequence is shown in red. (C) Schematic of the single guide RNA (sgRNA) targeting sites in pre-miR-155. The locations of the two arrows indicate the loci targeted by sgRNA1 and sgRNA2. Using the CRISPR/Cas9 system, two different sgRNAs simultaneously targeted from a single construct were directed to induce precise mutations in the mouse genomic loci.
(TIF)

S2 Fig A miR-155 knockout mouse model was established using the CRISPR/Cas9 system. (A) Three genotypes of F0 mice. The targeted sequences are shown in red. Three different mutations were found: a 1-bp deletion (D1), 1-bp insertion (I1) and a 114-bp deletion (KO). (B) Agarose gel electrophoresis of the F0 mice. A 472-bp fragment was PCR amplified, cloned and sequenced. The sequence alignments and chromatogram showed a 114-bp deletion in the KO mice. The expected fragment size was WT = 472 bp and KO (gene knockout) = 358 bp. (C) Sequences from F0 mice obtained by gene sequencing of the PCR products. Gene sequencing of the PCR products confirmed that the 114-bp gene fragment that was lost included the miR-155 host gene (HG) sequence. The successfully knocked out 114 bp fragment contained the 65 nucleotide stem-loop sequence of miR-155.
(TIF)

S3 Fig Establishment of the mouse line. (A) Representative photos of the KO (-/-), WT (+/+) and heterozygous (+/-) mice. (B) Establishment of the genotype by agarose gel electrophoresis. The presence of 2 bands on the gel indicates a heterozygous (+/-) genotype, a single higher-weight band indicates a wild-type (+/+) genotype, and a single lower-weight band indicates a homozygous (-/-) (KO) genotype.
(TIF)

S4 Fig Genotypes of all mice were distinguished by agarose gel electrophoresis. (TIF)

S1 Table List of abbreviations. (PDF)
