# Supplementary figures and images for: A comparative study of the characterization of miR-155 in knockout mice
Source: PLoS One. 2017 Mar 9;12(3):e0173487. doi: 10.1371/journal.pone.0173487 (PMC5344489; doi:10.1371/journal.pone.0173487)

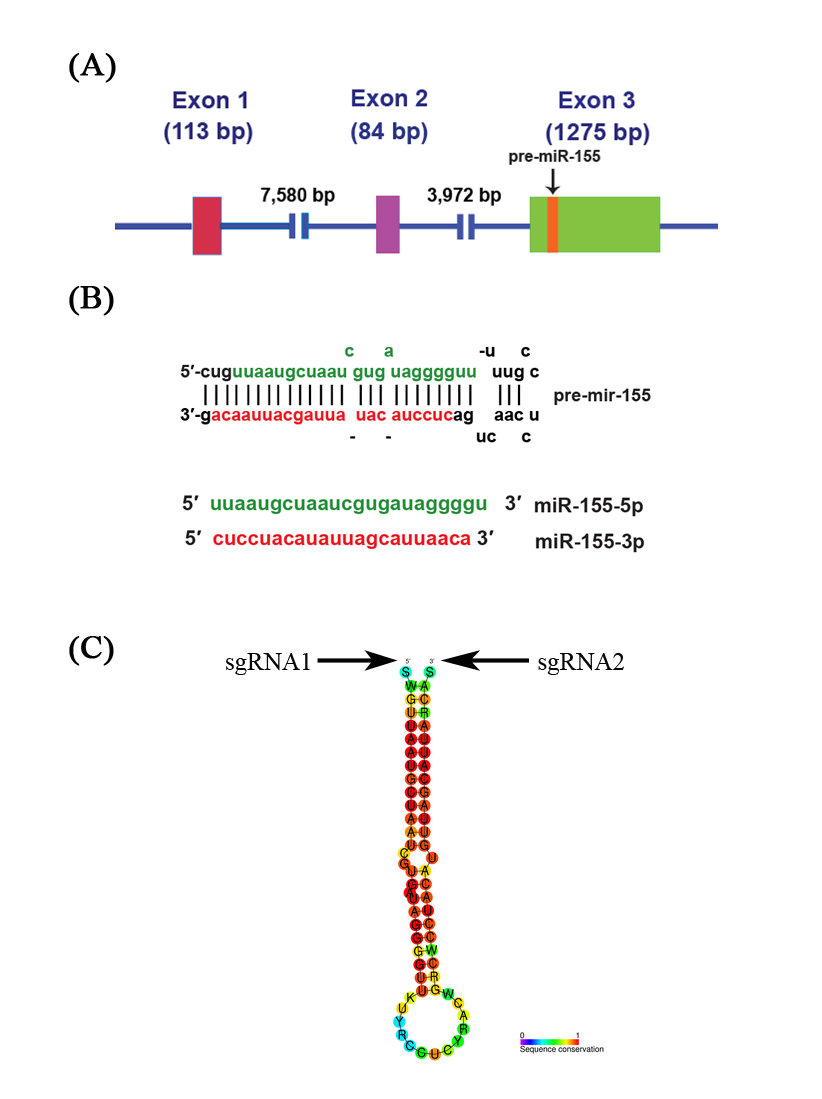

Supplement: S1 Fig — miR-155 is encoded by the miR-155 host gene (HG). The miR-155 HG is processed into two pre-microRNAs (miR-155-5p and miR-155-3p). (A) This gene is composed of three exons, spans 13,024 bp, and encodes a 1500-bp non-coding primary-miRNA (pri-miRNA). Pre-miR-155 is matured from the pri-miRNA transcript. The location of pre-miR-155 is indicated by the orange box. (B) Pre-miR-155 includes a base-paired stem loop. The miR-155-5p sequence is shown in green, and the miR-155-3p sequence is shown in red. (C) Schematic of the single guide RNA (sgRNA) targeting sites in pre-miR-155. The locations of the two arrows indicate the loci targeted by sgRNA1 and sgRNA2. Using the CRISPR/Cas9 system, two different sgRNAs simultaneously targeted from a single construct were directed to induce precise mutations in the mouse genomic loci. (TIF) [file pone.0173487.s001.tif]

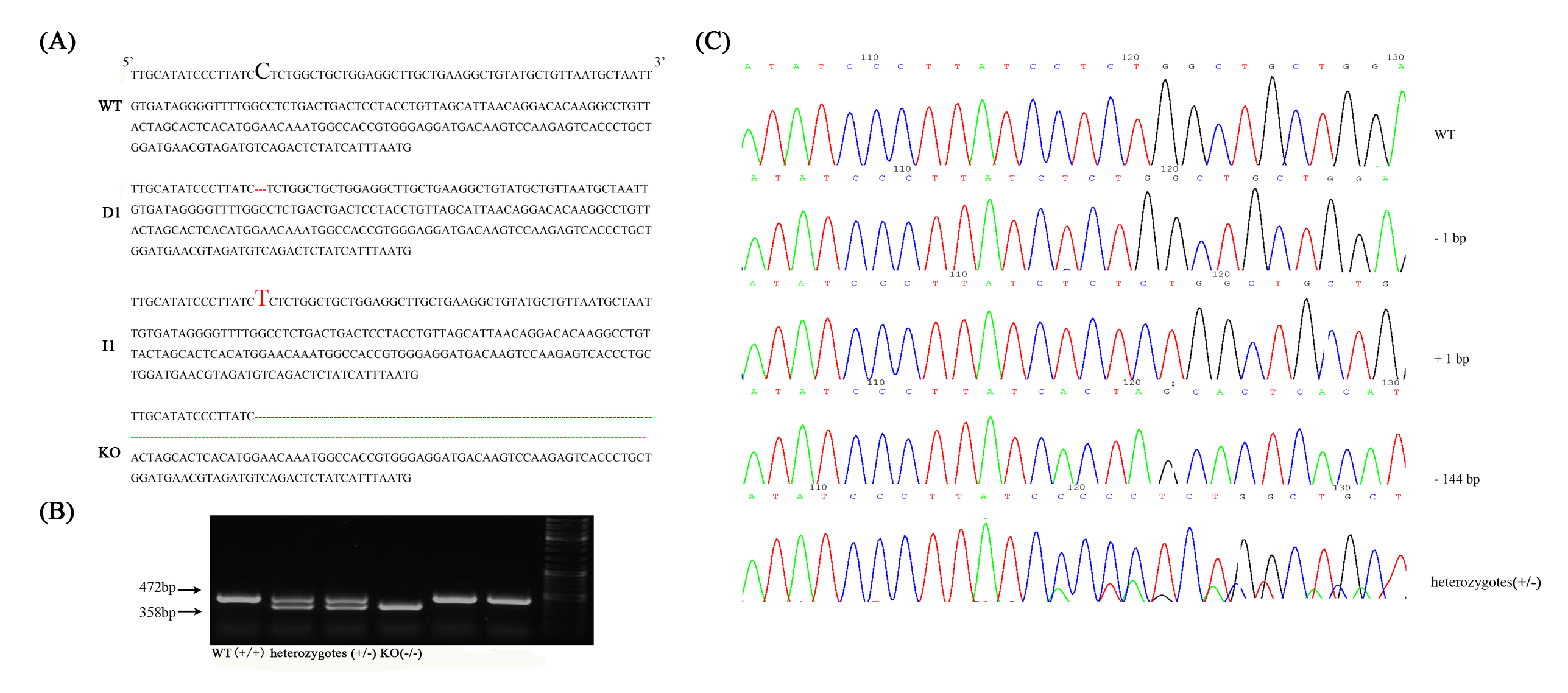

Supplement: S2 Fig — (A) Three genotypes of F0 mice. The targeted sequences are shown in red. Three different mutations were found: a 1-bp deletion (D1), 1-bp insertion (I1) and a 114-bp deletion (KO). (B) Agarose gel electrophoresis of the F0 mice. A 472-bp fragment was PCR amplified, cloned and sequenced. The sequence alignments and chromatogram showed a 114-bp deletion in the KO mice. The expected fragment size was WT = 472 bp and KO (gene knockout) = 358 bp. (C) Sequences from F0 mice obtained by gene sequencing of the PCR products. Gene sequencing of the PCR products confirmed that the 114-bp gene fragment that was lost included the miR-155 host gene (HG) sequence. The successfully knocked out 114 bp fragment contained the 65 nucleotide stem-loop sequence of miR-155. (TIF) [file pone.0173487.s002.tif]

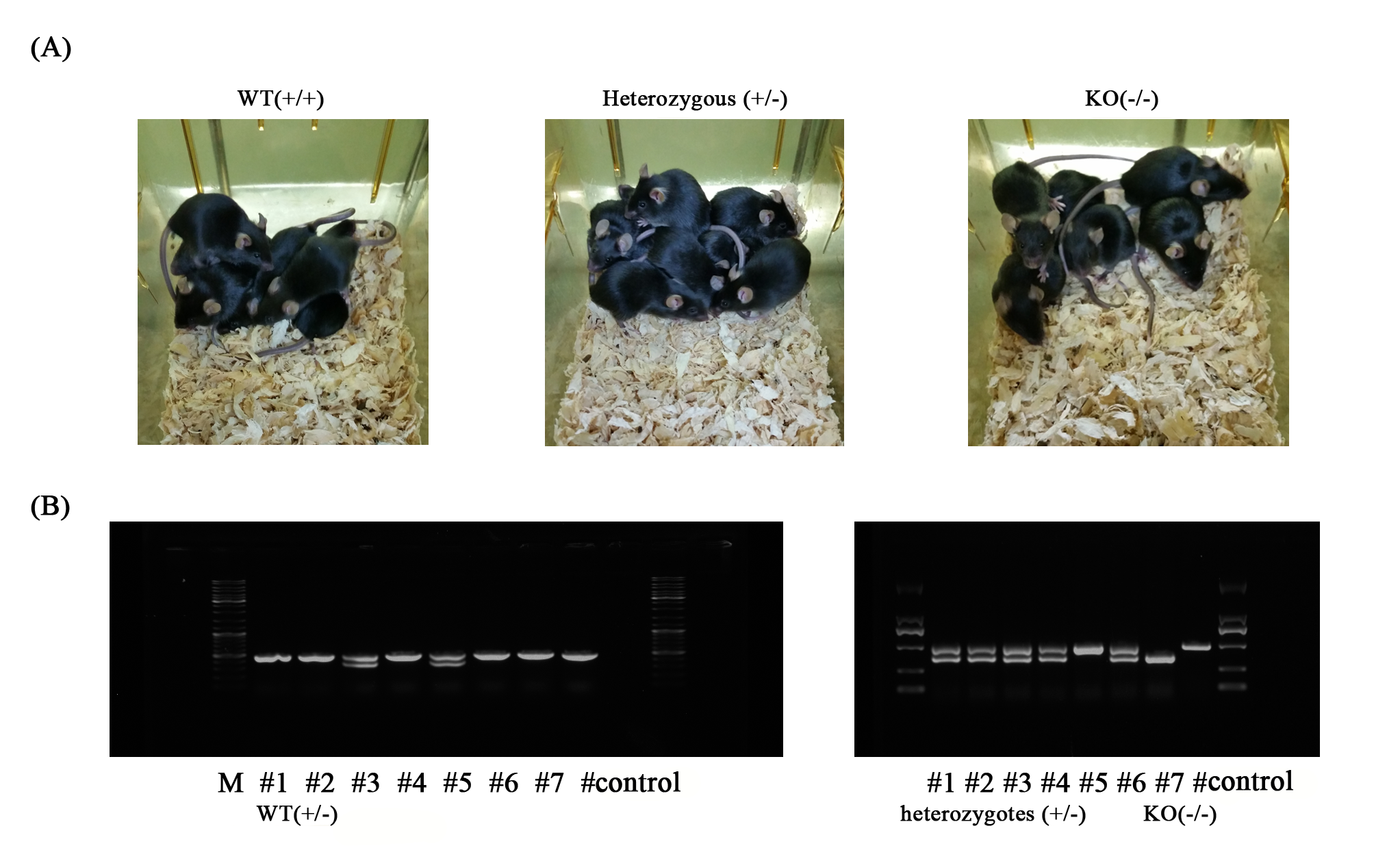

Supplement: S3 Fig — (A) Representative photos of the KO (-/-), WT (+/+) and heterozygous (+/-) mice. (B) Establishment of the genotype by agarose gel electrophoresis. The presence of 2 bands on the gel indicates a heterozygous (+/-) genotype, a single higher-weight band indicates a wild-type (+/+) genotype, and a single lower-weight band indicates a homozygous (-/-) (KO) genotype. (TIF) [file pone.0173487.s003.tif]

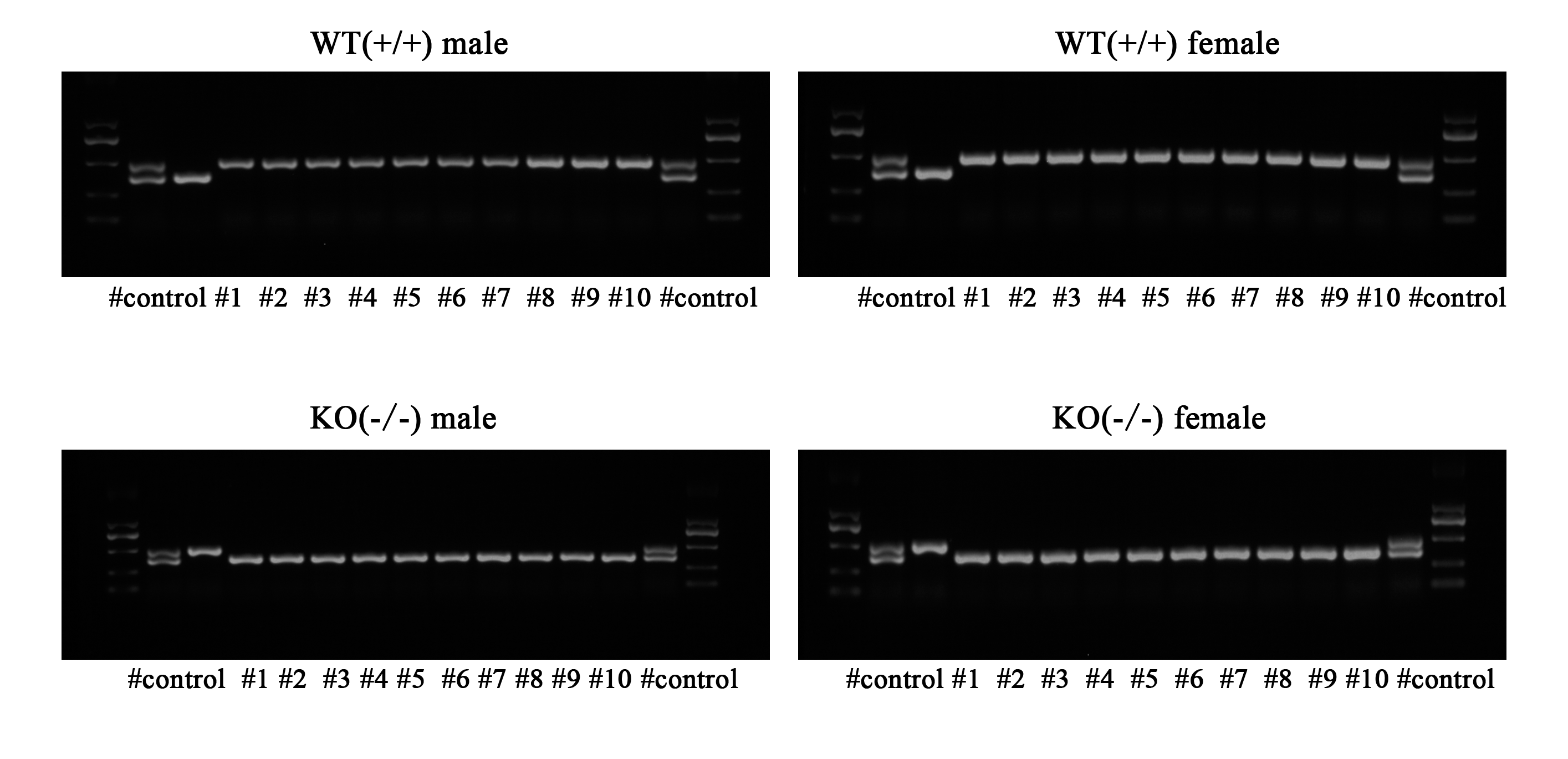

Supplement: S4 Fig — (TIF) [file pone.0173487.s004.tif]
