# Supplementary material for: A comparative study of the characterization of miR-155 in knockout mice
Source: PLoS One. 2017 Mar 9;12(3):e0173487. doi: 10.1371/journal.pone.0173487 (PMC5344489; doi:10.1371/journal.pone.0173487)
Supplement: S1 Table — (PDF) [file pone.0173487.s005.pdf]

S1Table. List of abbreviations

A/G	albumin/ globulin
ALB	Albumin
ALT	Alanine aminotransferase
AST	Aspartic transaminase
BAS	Basophils
BUN	Blood urea nitrogen
CO	Cardiac output
CRE	Creatinine
EOS	Eosinophils
GLB	globulin
GLU	Blood glucose
HCT	Hematocrit
HGB	Hemoglobin
IVS	Interventricular septum
LA	Left atrium
LV	Left ventricle
LVAW(D)	Left ventricular anterior wall (Diastole)
LVAW(S)	Left ventricular anterior wall (Shrink)
LVEDD	Left ventricular end-diastolic diameter
LVEDV	left ventricular end-diastolic volume
LVEF	Left ventricular ejection fraction
LVESD	Left ventricular end-systolic diameter
LVESV	Left ventricular end-systolic volume
LVFS	Left ventricular fractional shortening
LVM	LV mass
LVOT	Left ventricular outflow tract
LVPW(D)	Left ventricular posterior wall (Diastole)
LVPW(S)	Left ventricular posterior wall (Shrink)
LVSV	Left ventricular stroke volume
LYM	Lymphocytes
MCH	Mean corpuscular hemoglobin
MCHC	Mean corpuscular hemoglobin concentration
MCV	Mean corpuscular volume
Mean Vel	Mean velocity
MON	Monocytes

MPV	Mean platelet volume
NEUT	Neutrophils
PCT	Thrombocytocrit
PDW	Platelet distribution width
Peak Vel	Peak velocity
PLT	Platelets
PV	Portal vein
PV Diam	Portal vein diameter
RA	Renal artery
RA Diam	Renal artery diameter
RAEDV	Renal artery end diastolic velocity
RAPSV	Renal artery peak systolic velocity
RARI	Renal artery resistance index
RBC	Red blood cells
RDW	Red cell distribution width
TP	Total protein
VTI	Velocity time integral
WBC	White blood cells
